# High-resolution multi-breath-held 3D volumetric T1 mapping acquisition: analysis of T1 measurement reproducibility compared to 2D T1 mapping with a respiratory motion phantom

**DOI:** 10.1186/1532-429X-17-S1-O2

**Published:** 2015-02-03

**Authors:** Keigo Kawaji, Sui-Cheng Wang, Akiko Tanaka, Hui Wang, Roberto Lang, Amit R Patel

**Affiliations:** 1 Medicine, Section of Cardiology, The University of Chicago, Chicago, IL, USA; 2 Medicine, Section of Surgery, The University of Chicago, Chicago, IL, USA; 3 Biomedical Engineering, Northwestern University, Chicago, IL, USA; 4 Philips Healthcare, Cleveland, OH, USA

## Background

Myocardial T1 mapping, which is used to detect diffuse fibrosis and quantify extracellular volumes, often employs 8-10 mm 2D slice acquisitions. However, this may be unsuited for accurate quantification of small tissues samples. We propose a new methodology that improves through-plane resolution using a novel 3D acquisition technique over multiple breath-holds. In this study, we compared this new approach to the reference multi-slice 2D approach using a respiratory motion phantom.

## Methods

**Figure 1:**
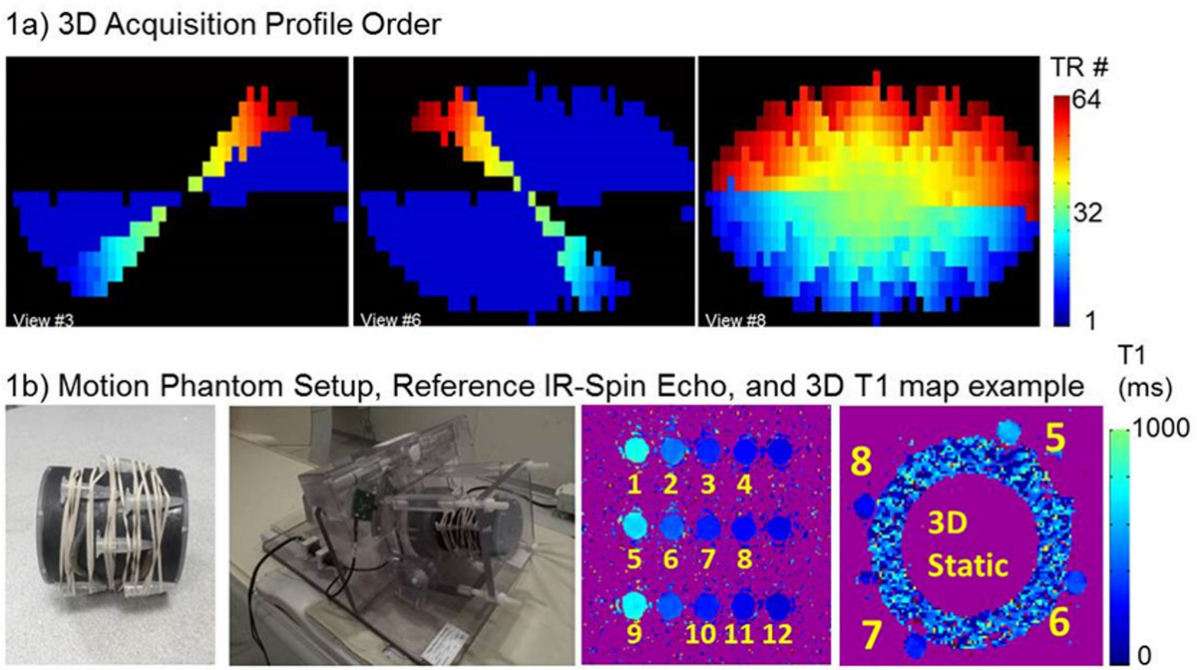
a) Profile ordering of the proposed 3D method. b) Motion phantom and T1 maps from the reference IR-SE and from the 3D sequence, respectively

The proposed 3D sequence employs a Cartesian projection of radial sectors, where each opposite sector pair is acquired in a single readout crossing through the center of k-space at the acquisition window midpoint (Fig 1a). Spatial resolution is gained in 3D via partial kz (~62%) and circular shutter Field-of-View (FOV) (~27% reduction), yielding ~3x improvement in through-plane resolution.

Imaging was performed using 1.5T MRI (Philips Achieva) with a 4-channel array on a respiratory motion phantom with 12 conical vials with varying Gd concentrations (fig 1b). The proposed 3D sequence employed a 5-(3s)-3 Modified Look-Locker Inversion Recovery (MOLLI) over ~11 heart beats per breath-hold. 2D MOLLI was also acquired across the same FOV. The following breathing patterns were examined: 1) no respiratory motion, 2) 8mm respiratory drift, and 3) 4-8 mm respiratory shifts between alternating breath-holds. 1 and 2 were repeated 5x for reproducibility, and 3 was implemented 4x to introduce motion blurring in the 3D approach. The following parameters were used for both 2D and 3D MOLLI: FOV (185x185x80mm), # of breath-holds (n=8), resolution (1.7x2.1 mm) with 2D vs 3D slice thickness/resolution = 10 vs 3.1 mm (8 and 26 slices, respectively). SENSE with R=2 acceleration was also used. A reference IR-SE scan was used to determine the reference T1 values for each vial. Relative T1 error of the 2D and 3D MOLLI measurements was calculated as ∆T1 = 100*|T1_meas_-T1_Ref_|/T1_Ref_. All measurements were performed using custom software. Student's t-test was used to compare inter-technique T1 measurements (p<0.05 significant).

## Results

Both static 2D and 3D MOLLI yielded comparable (p=0.4) relative error with respect to the reference IR-SE T1 measurements. Under an 8mm mid-breath-hold drift, 2D MOLLI was unable to yield T1 values in 5 out of the 12 vials (42%), while 3D MOLLI allowed T1 measurement of all vials. The variation in the average T1 measurements across the 5 repeated scans was less with the 3D approach (2D vs 3D: 18.5±7 vs 8.4±6 ms; p<0.05). T1 mapping was feasible even under motion blurred 3D volumes, and both measured T1 values and T1 error were comparable to those from static 3D volumes (p=0.5, p=0.3, respectively).

## Conclusions

Respiratory motion can result in failure of conventional 2D T1 mapping. The proposed novel 3D T1 mapping scheme performs robustly in the presence of respiratory motion at a significantly higher spatial resolution.

## Funding

N/A.

**Table 1:** Relative T1 Error: 100*|T1_meas_ - T1_IR-SE_ |/T1_IR-SE_

T1_meas_	% Error vs Ref IR-SE	P-value
2D Static	9.8 ± 4.8	P = NS

2D with Respiratory Drift	10.0 ± 5.1	P = NS

2D with Respiratory Shift	11.7 ± 7.7	P = NS

3D Static	7.6 ± 6.1	P = NS

3D with Respiratory Drift	9.6 ± 9.3	P = NS

3D with Respiratory Shift	9.1 ± 9.2	P = NS

All pairwise comparisons yielded non-significant (NS) p-values (P > 0.05).

